# Development and internal validation of predictive models to assess risk of post-acute care facility discharge in adults undergoing multi-level instrumented fusions for lumbar degenerative pathology and spinal deformity

**DOI:** 10.1007/s43390-022-00582-w

**Published:** 2022-09-20

**Authors:** Ayush Arora, Joshua Demb, Daniel D. Cummins, Vedat Deviren, Aaron J. Clark, Christopher P. Ames, Alekos A. Theologis

**Affiliations:** 1 Department of Orthopaedic Surgery, University of California-San Francisco (UCSF), 500 Parnassus Ave, MUW 3rd Floor, San Francisco, CA 94143 USA; 2 Division of Gastroenterology, Department of Medicine, University of California-San Diego, La Jolla, CA USA; 3 Department of Neurological Surgery, UCSF, San Francisco, CA USA

**Keywords:** Adult spinal deformity, Thoracolumbar fusion, Discharge, Acute rehabilitation, Predictive modeling

## Abstract

**Purpose:**

To develop a model for factors predictive of Post-Acute Care Facility (PACF) discharge in adult patients undergoing elective multi-level (≥ 3 segments) lumbar/thoracolumbar spinal instrumented fusions.

**Methods:**

The State Inpatient Databases acquired from the Healthcare Cost and Utilization Project from 2005 to 2013 were queried for adult patients who underwent elective multi-level thoracolumbar fusions for spinal deformity. Outcome variables were classified as discharge to home or PACF. Predictive variables included demographic, pre-operative, and operative factors. Univariate and multivariate logistic regression analyses informed development of a logistic regression-based predictive model using seven selected variables. Performance metrics included area under the curve (AUC), sensitivity, and specificity.

**Results:**

Included for analysis were 8866 patients. The logistic model including significant variables from multivariate analysis yielded an AUC of 0.75. Stepwise logistic regression was used to simplify the model and assess number of variables needed to reach peak AUC, which included seven selected predictors (insurance, interspaces fused, gender, age, surgical region, CCI, and revision surgery) and had an AUC of 0.74. Model cut-off for predictive PACF discharge was 0.41, yielding a sensitivity of 75% and specificity of 59%.

**Conclusions:**

The seven variables associated significantly with PACF discharge (age > 60, female gender, non-private insurance, primary operations, instrumented fusion involving 8+ interspaces, thoracolumbar region, and higher CCI scores) may aid in identification of adults at risk for discharge to a PACF following elective multi-level lumbar/thoracolumbar spinal fusions for spinal deformity. This may in turn inform discharge planning and expectation management.

**Supplementary Information:**

The online version contains supplementary material available at 10.1007/s43390-022-00582-w.

## Introduction

Adult spinal deformity (ASD) is a disabling health state associated with poorer health-related quality of life (HRQOL) and greater functional deficits when compared to other chronic illnesses [1, 2]. While operative intervention, consisting of multi-level lumbar/thoracolumbar posterior instrumented fusions, can provide meaningful improvement of functional status, recovery can be arduous [3]. As such, rehabilitation is commonly a critical component of care for patients with ASD following surgery. Discharge to a post-acute care facility (PACF) is often necessary given the need for extended acute care, lack of social support, and management of peri-operative complications [4, 5]. The benefits of rehabilitation include offering improved mental health, improved function, and earlier return to work compared to patients who do not undergo rehabilitation [6, 7]. However, financial planning, quality, and efficiency surrounding the transition to rehabilitation care suffer from many gaps. Discharge to a PACF is associated with a high cost for ASD patients, accounting for approximately 30% of care costs [8]. Inpatient delays in discharge referral to a PACF can lead to longer hospital lengths of stay (LOS) and time lost for recovery [9, 10]. Significant administrative capacity is also needed to obtain a referral for rehabilitation and complete the transfer process.

Identification of ASD patients pre-operatively at increased risk for discharge to a PACF holds the purported benefits of improving pre-operative planning, reducing hospital LOS through early administrative action, and management of patient expectations [11, 12]. While some studies have identified certain risk factors associated with discharge to a PACF for ASD patients, limited cohort sizes have hampered their ability to develop robust, data-driven prediction models [13, 14]. Moreover, conflicting importance placed on certain risk factors adds difficulty to risk assessment. As such, the purpose of this study is to develop and internally validate a predictive model that utilizes patient risk factors to generate a pre-operative likelihood of PACF discharge in adults undergoing elective, multi-level lumbar/thoracolumbar operations for lumbar pathology, and spinal deformity.

## Methods

### Source of data

A retrospective review of state-level inpatient databases was conducted within the Healthcare Cost and Utilization Project (HCUP) to examine predictive factors for discharge to a PACF among adults with ASD [15]. HCUP is composed of numerous healthcare databases sponsored by the Agency for Healthcare Research and Quality, boasting the largest collection of longitudinal hospital care data in the United States [16]. The State Inpatient Databases contains inpatient discharge data from both academic and private tertiary care centers in California, Florida, Nebraska, New York, North Carolina, and Utah. Our study incorporates state inpatient database data from 2005 to 2013.

### Participants, sample size, and missing data

**Fig. 1:**
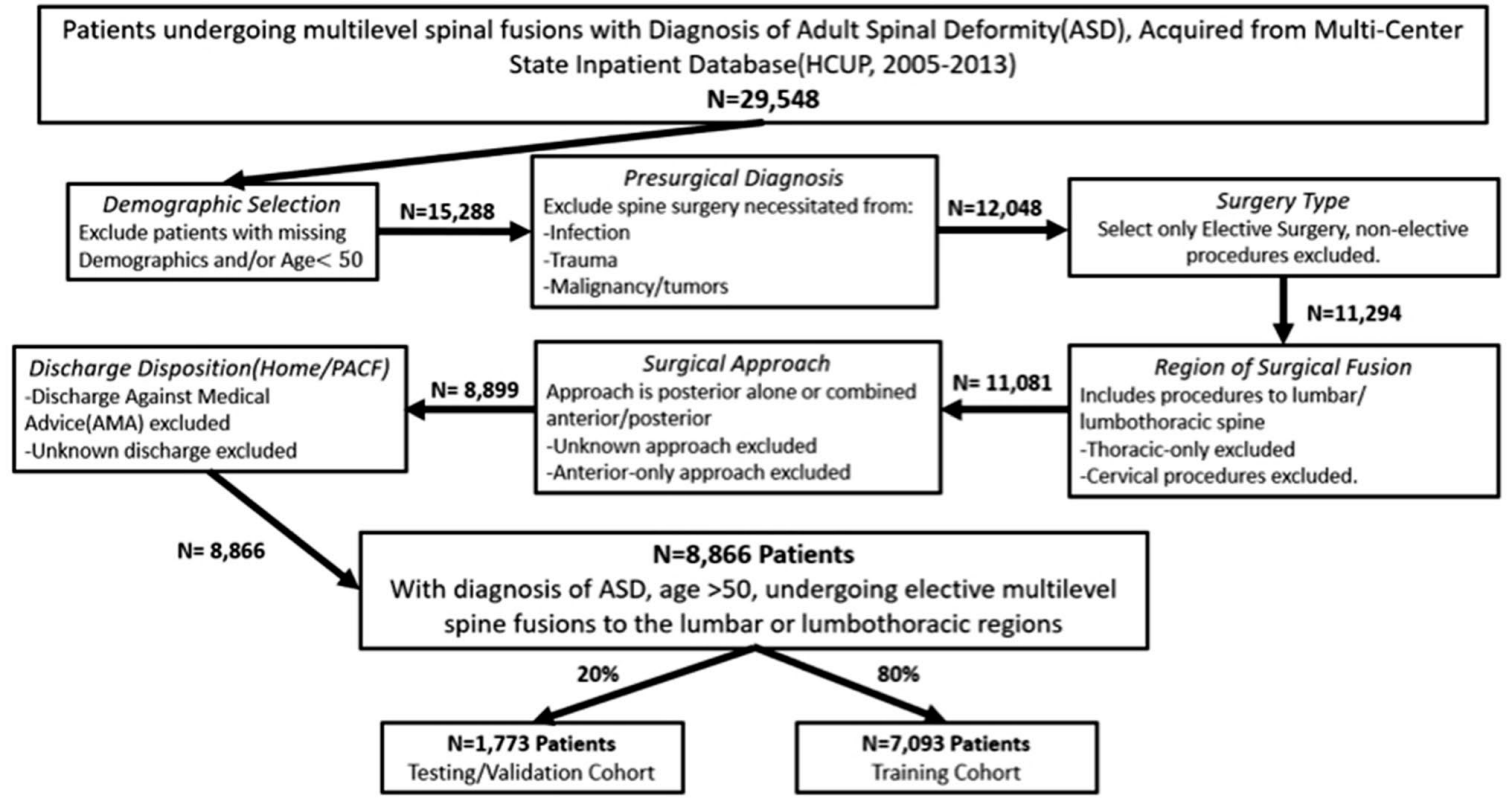
Patient selection flowchart

Eligibility criteria included adults ages ≥ 50 with prior a diagnosis of ASD, undergoing elective multi-level spine fusions, defined as instrumented fusions of $\ge$ 3 levels, in the lumbar or thoracolumbar regions (Fig. 1). Exclusion criteria included: age < 50 years, operations for infection, trauma, and/or malignancy, discharges against medical advice, and any missing predictor or outcome variables. Inclusion and exclusion criteria derived from International Classification of Diseases, Volumes 9 codes (ICD-9), were based on algorithms derived from prior literature [17–19]. While the state inpatient database of spine procedures consisted of 29,584 patients, the total study size was 8866 following eligibility criteria application.

### Predictors and outcomes

The primary study outcome was discharge to PACF after elective multi-level spinal fusion surgery (yes/no). Demographic, medical history, and operative variables were identified as potential predictors of discharge to PACF (Table 1). All predictors were measured prior to surgery, with medical history and operative variables identified by ICD-9 codes. Demographic variables included age (50–59, 60–69, 70–79, 80+), sex, race/ethnicity (Non-Hispanic White, Black or African American, Hispanic, Asian, Native American/Other), and healthcare institution type (Academic vs. Non-Academic). Insurance status was captured in categories of public (Medicare/Medicaid), private (Commercial), and other (Self-Pay/Other). Medical variables included comorbid health conditions, substance abuse (alcohol abuse, drug abuse, and smoking history), malnutrition, osteoporosis, and mental health conditions (anxiety and depression) [20]. The Charlson Comorbidity Index (CCI) score (0, 1, 2, 3, or ≥ 4) was also measured. Operative variables included surgical approach (posterior, combined anterior, and posterior), region of surgery (lumbar only vs. lumbar and thoracic), revision surgery (yes/no), and vertebral levels fused and instrumented (3–7 levels vs. ≥ 8 levels).

**Table 1:** Baseline data

Variable	Discharge to home (%)	Discharge to PACF (%)	*p*
Population	4904 (55.3%)	3962 (44.7%)	–
Age (median, Q1, Q3)	65 (58,71)	71 (65,77)	–
50–59	1393 (75.4%)	454 (24.6%)	Ref
60–69	1987 (61.0%)	1272 (39.0%)	< 0.01
70–79	1271(43.7%)	1638 (56.3%)	< 0.01
≥ 80	253(29.7%)	598 (70.3%)	< 0.01
Gender			–
Male	1784 (63.0%)	1048 (37.0%)	Ref
Female	3120 (51.7%)	2914 (48.3%)	< 0.01
Race			–
White	4197 (55.6%)	3347 (44.4%)	Ref
Hispanic	208 (49.5%)	212 (50.5%)	0.02
Black	102 (48.3%)	109 (51.7%)	0.04
Asian	49 (49.5%)	50 (50.5%)	0.22
Native American/other	348 (58.8%)	244 (41.2%)	0.83
Surgical approach			–
Posterior	3891 (55.8%)	3079 (44.2%)	Ref
Anterior and posterior (combined)	1013 (53.4%)	883 (46.6%)	0.07
Region			–
Lumbar only	4144 (57.4%)	3074 (42.6%)	Ref
Lumbar and thoracic	760 (46.1%)	888 (53.9%)	< 0.01
Revision	1182 (59.6%)	801 (40.4%)	< 0.01
# Levels instrumented/fused			–
3–7 Levels	4464 (56.9%)	3377 (43.1%)	Ref
≥ 8 Levels	440 (42.9%)	585 (57.1%)	< 0.01
Institutional type			–
Non-academic	3841 (56.0%)	3023 (44.0%)	Ref
Academic	708 (49.9%)	710 (50.1%)	< 0.01
Insurance type			–
Public	2646 (46.2%)	3081 (53.8%)	Ref
Private	1891 (72.8%)	705 (27.2%)	< 0.01
Other	367 (67.6%)	176 (32.4%)	< 0.01
Charlson’s comorbidity index (CCI)			–
CCI = 1	222 (20.8%)	844 (79.2%)	Ref
CCI = 2	645 (31.1%)	1428 (68.9%)	< 0.01
CCI = 3	1155 (47.0%)	1303 (53.0%)	< 0.01
CCI ≥ 4	1940 (59.3%)	1329 (40.7%)	< 0.01
Co-morbidities			
Chronic pulmonary disease	1021 (50.1%)	1016 (49.9%)	< 0.01
Congestive heart disease (CHF)	206 (42.0%)	285 (58.0%)	< 0.01
Hemiplagia/paraplegia	81 (40.3%)	120 (59.7%)	< 0.01
Past myocardial infarction	275 (48.6%)	291 (51.4%)	< 0.01
Renal disease	177 (40.3%)	262 (59.7%)	< 0.01
Rheumatic disease	307 (48.3%)	329 (51.7%)	< 0.01
Hypertension	3028 (52.1%)	2781 (47.9%)	< 0.01
Malnutrition	56 (34.6%)	106 (65.4%)	< 0.01
Coronary artery disease (CAD)	749 (48.9%)	783 (51.1%)	< 0.01
Hypothyroidism	830 (49.2%)	856 (50.8%)	< 0.01
Osteoporosis	581 (43.2%)	763 (56.8%)	< 0.01
Diabetes			
No diabetes	4153 (57.0%)	3129 (43.0%)	Ref
Controlled diabetes	695 (47.3%)	775 (52.7%)	< 0.01
Uncontrolled diabetes	56 (49.1%)	58 (50.9%)	0.11
Substance abuse			
Smoking history	1600 (57.6%)	1178 (42.4%)	< 0.01
Alcohol abuse	108 (52.2%)	99 (47.8%)	0.36
Drug abuse	128 (48.5%)	136 (51.5%)	0.03
Mental health			
Anxiety	579 (54.5%)	483 (45.5%)	0.60
Depression	1053 (51.2%)	1003 (48.8%)	< 0.01

*PACF* post-acute care facility

### Statistical analysis

Univariate analysis through Fisher’s exact test and binary logistic regression were used to examine associations between potential predictors and PACF discharge, deriving odds ratios (OR), and 95% confidence intervals (CI). Predictors showing a *p* value less than 0.05 or 95% CI not crossing unity (OR = 1.0) were included in three predictive models: fully saturated multivariable logistic regression, decision tree learning, and Bayesian predictive modeling.

### Development and validation of predictive models

Prediction modeling was conducted by splitting cohort into 80% derivation and 20% validation cohorts. Area under the receiver-operating curve (AUC) for each model with corresponding 95% CIs was calculated to compare diagnostic performance. Since development and validation groups were derived from the same dataset, both groups utilized the same eligibility criteria, outcome measure, and predictors.

To create a simplified prediction model with similar diagnostic performance to the multivariable logistic regression model, we used a priori literature review and a least absolute shrinkage and selection operator (LASSO) model to choose variables of greatest importance. Variables with greatest significance were added in stepwise manner to derive the model with the best diagnostic performance, measured using the Receiver-Operating Curve (ROC). Stepwise model creation included variables with *p* value less than 0.05 upon inclusion until diagnostic performance did not change by more than 0.5%. Upon completion of the prediction model, AUC was calculated, as well as diagnostic characteristics including sensitivity, specificity, positive predictive value, and negative predictive value. A calibration curve of the final predictive model was also developed to examine how predicted PACF discharge compared to observed PACF discharge. A Brier score was determined to quantify the accuracy of the probabilistic predictions. The Brier score is a quantifiable evaluation metric (ranging from 0 to 1) determined by calculating the sum of the mean-squared probability errors, divided by the total number of predictions generated. Lower Brier scores (closer to 0) indicate better model forecasting ability. To maximize discrimination of the predictive model, different predictive probability cut-offs and associated diagnostic characteristics were compared to choose a final model cut-off at which sensitivity and specificity were maximized for discharge to PACF. We used MATLAB version 2020b to conduct analyses [21].

## Results

### Participants

Among the 8866 patients who met the inclusion criteria, 55.3% were discharged home and 44.7% were discharged to a PACF (Table 1). The median age was 71 years (Q1–Q3: 65–77) and female patients represented 68.1% of the cohort. The majority of patients had a CCI score of at least three (64.6%). The most common comorbidities were hypertension (65.5%) and smoking history (31.8%). Most operations were restricted to the lumbar spine (81.4%) and consisted of a posterior-only approach (78.6%).

### Univariate and multivariate analyses (Table 2)

**Table 2:** Multivariate analysis

Variable	OR	95% CI	*p*
Age (continuous)			
50–59	–	*–*	–
60–69	1.51	1.24–1.84	< 0.001
70–79	2.45	1.90–3.14	< 0.001
≥ 80	4.53	3.29–6.23	< 0.001
Gender			
Female	–	–	–
Male	0.64	0.57–0.71	< 0.001
Race			
White	–	–	–
Hispanic	1.22	0.98–1.51	0.072
Black	1.76	1.30–2.36	< 0.001
Asian	–	–	–
Native American/other	–	–	–
Region			
Lumbar only	*–*	–	–
Lumbar and thoracic	1.63	1.41–1.87	< 0.001
Revision surgery	0.65	0.57–0.73	< 0.001
Vertebral levels			
3–7 levels	*–*	–	–
≥ 8 levels	1.64	1.39–1.94	< 0.001
Institutional type			
Non-academic	*–*	–	–
Academic	1.41	1.24–1.60	< 0.001
Insurance type			
Public	*–*	–	–
Private	0.57	0.50–0.64	< 0.001
Other	0.78	0.63–0.96	0.017
Charlson’s comorbidity index (CCI)			
CCI = 1	*–*	–	–
CCI = 2	1.08	0.85–1.38	0.515
CCI = 3	1.32	1.00–1.75	0.052
CCI ≥ 4	1.43	1.02–2.01	0.040
Co-morbidities			
Chronic pulmonary disease	1.16	1.02–1.33	0.027
Congestive heart failure (CHF)	1.15	0.93–1.41	0.200
Hemiplegia/paraplegia	1.59	1.15–2.19	0.005
Past myocardial infarction	1.08	0.88–1.34	0.453
Renal disease	1.31	1.05–1.63	0.018
Rheumatic disease	1.01	0.84–1.22	0.882
Hypertension	1.14	1.03–1.26	0.014
Malnutrition	1.73	1.22–2.47	0.002
Coronary artery disease (CAD)	0.95	0.82–1.09	0.457
Hypothyroidism	1.02	0.90–1.14	0.835
Osteoporosis	1.21	1.07–1.38	0.004
Controlled diabetes	1.27	1.10–1.46	0.001
Substance abuse			
Smoking history	0.91	0.81–1.00	0.051
Drug abuse	1.57	1.20–2.06	0.001
Mental health			
Depression	1.31	1.17–1.46	< 0.001

Results for the univariate (Table 1) and multivariate logistic regression analyses (Table 2) demonstrated the following pre-operative factors as significant risks for PACF discharge: age ≥ 60 years, African American race, increased CCI scores, COPD, hypertension, hemiplegia/paraplegia, renal disease, drug abuse, osteoporosis, depression, controlled diabetes mellitus, and academic institution. Operative factors associated with increased risk of a PACF discharge were longer fusions involving both the thoracic and lumbar spine (≥ 8 levels). Private insurance, male gender, and revision procedures decreased risk of PACF discharge.

### Model development

**Fig. 2:**
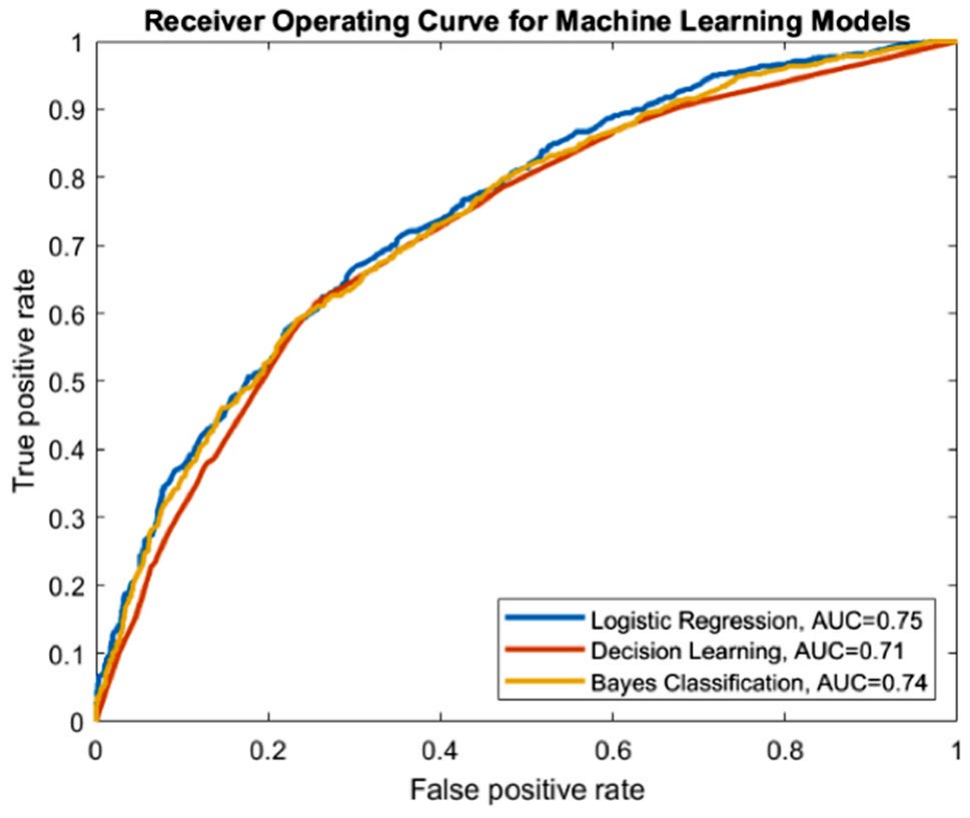
Receiver-operating curve (ROC) for logistic regression, decision learning, and Bayes classification predictive models for discharge disposition to either home or PACF. The AUCs were 0.75 (95% CI 0.73–0.77), 0.71 (95% CI 0.67–0.73), and 0.74 (95% CI 0.72–0.76), respectively

Using the significant variables derived from multivariate analysis, three predictive models were developed (Fig. 2): multivariable logistic regression (AUC = 0.75, 95% CI 0.73–0.77), decision tree learning (AUC = 0.71, 95% CI 0.67–0.73), and Bayesian classification (AUC = 0.74, 95% CI 0.72–0.76). Eighty percent of the cohort (*N* = 7093) was used in development of each model with validation on the remaining 20% (*N* = 1773).

### Model specification: creation of a simplified logistic predictive model

**Fig. 3:**
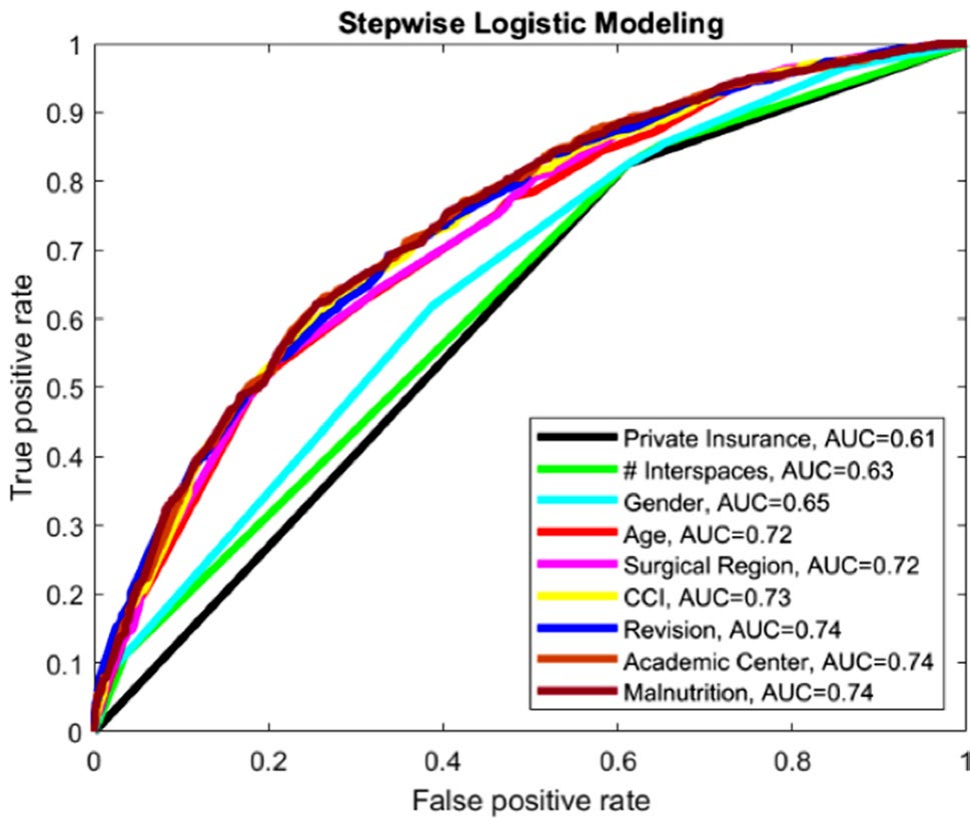
Stepwise logistic regression. Each curve represents a logistic predictive model using one additional variable. For example, the black curve represents a predictive model only using insurance (private), while the light-blue curve represents model using insurance (private), number of levels (8+), and gender (Male)

The nine most relevant variables identified via LASSO regression with the highest coefficients were as follows: private insurance, number of interspaces fused/instrumented, gender, age, region of surgery, CCI, revision surgery, type of institution, and malnutrition. As each variable was successively added to the model, the ROC was graphed and AUC was calculated (Fig. 3). Peak AUC was reached with seven of nine selected predictors. These seven predictors included private insurance, number of interspaces, gender, age, surgical region, CCI, and revision surgery. The addition of institution type and malnutrition increased AUC by less than 0.3% and were hence deemed unnecessary to reach peak AUC. The ORs and 95% CIs for each component of the simplified logistic predictive model were derived (Table 3), and beta coefficients were determined (Supplementary Table 1) for use in a predictive calculator.

**Table 3:** Odds ratios of final logistic regression model for PACF discharge

Logistic model component	OR	95% CI	*p*
Insurance (private)	0.58	0.51–0.66	< 0.001
# Interspaces (8+)	1.61	1.34–1.93	< 0.001
Gender (male)	0.62	0.55–0.69	< 0.001
Age^a^	1.38	1.27–1.50	< 0.001
Surgical region (lumbar + thoracic)	1.64	1.41–1.92	< 0.001
CCI^b^	1.36	1.27–1.46	< 0.001
Revision surgery	0.71	0.62–0.80	< 0.001

*CCI* Charlson comorbidity index

^a^Per decade increase

^b^Per one point increase

### Model performance

**Fig. 4:**
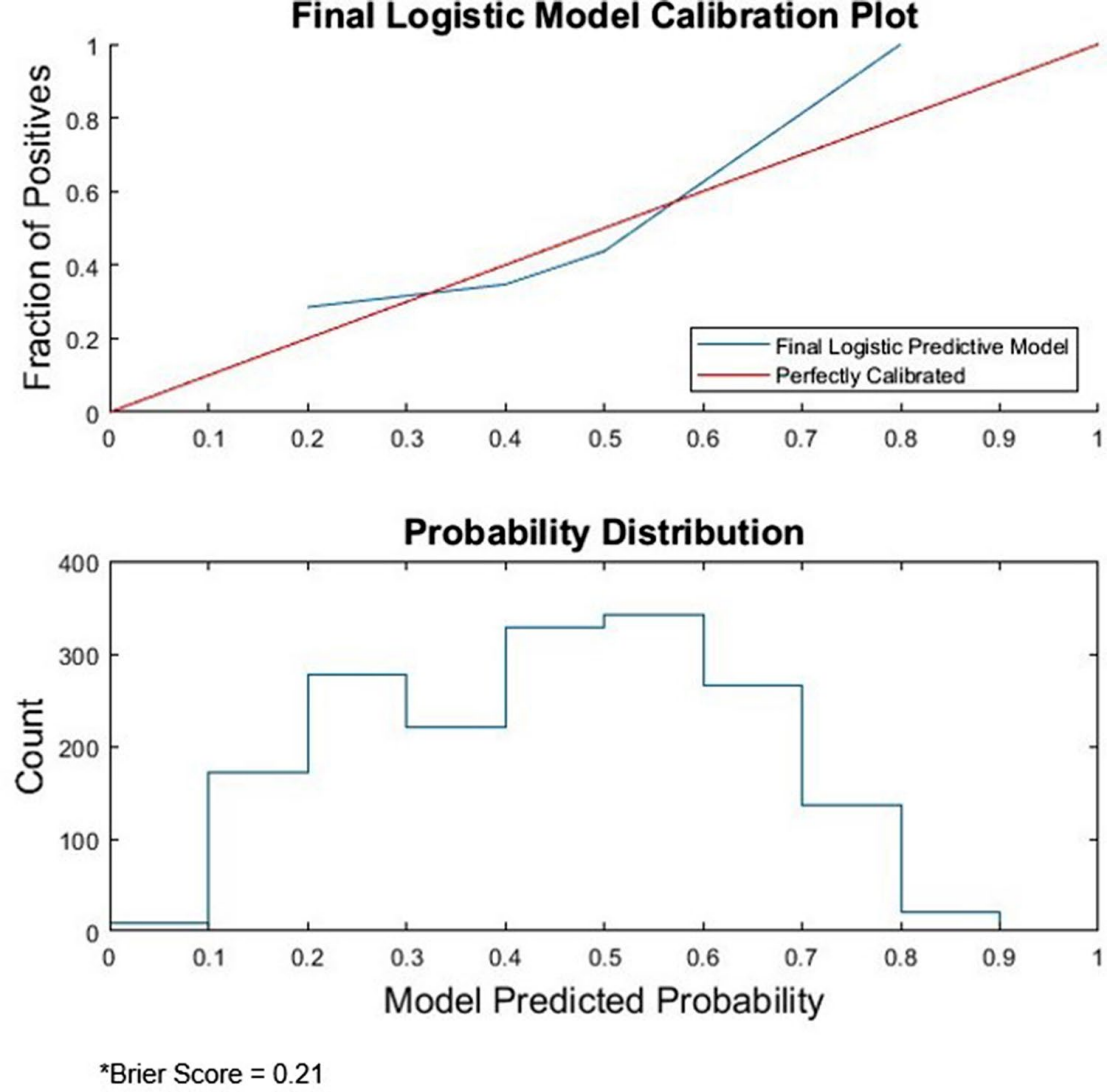
Calibration plot and probability distribution histogram of final predictive model

The final logistic model utilizing the seven selected predictors was validated on the remaining 20% of the cohort (*N* = 1773), producing an AUC of 0.74 (95% CI 0.72–0.76). Metrics, such as sensitivity, specificity, positive predictive value, and negative predictive value, for each threshold applied are displayed in Table 4. The calibration curve of the final predictive model reflects how the model overestimates predicted PACF discharge risk at moderate probabilities and underestimates PACF discharge risk at very low and high probabilities (Fig. 4). The Brier score of the final logistic model was 0.21.

**Table 4:** Predictive model characteristics depending on threshold level

Threshold	Sensitivity	Specificity	Positive predictive value	Negative predictive value
0.35	0.85	0.46	0.56	0.78
0.37	0.83	0.49	0.57	0.77
0.39	0.81	0.50	0.57	0.76
0.41	0.75	0.59	0.60	0.74
0.43	0.74	0.60	0.60	0.73
0.45	0.69	0.66	0.63	0.72
0.47	0.68	0.67	0.63	0.72
0.49	0.62	0.72	0.64	0.70
0.51	0.60	0.74	0.65	0.69
0.53	0.54	0.79	0.68	0.68
0.55	0.52	0.80	0.68	0.67

## Discussion

### Model interpretation

In this study, the goal was to identify significant pre-operative and operative factors associated with discharge to PACF facility following multi-level lumbar/thoracolumbar instrumented fusions for lumbar degenerative pathology and spinal deformity to develop a predictive calculator for clinical use. The predictive model, which utilized seven variables, presents a clinical tool that offers rapid pre-operative assessment of likely discharge location (home vs. PACF). With an AUC of 0.74, this prediction calculator has fair validity.

Based on our final model, we recommend a predicted probability cut-off of 0.41 to maximize diagnostic characteristics for PACF discharge predictions (sensitivity: 0.75, specificity: 0.59). A higher sensitivity (detection of patients who will be discharged to PACF) than specificity (detection of patients discharged home) may be more helpful than the opposite, as it would inform administrative teams and allow time to prepare for a PACF discharge pre-operatively and/or early in the post-operative setting. Preparing a patient for PACF discharge that is ultimately not needed (false positive) may be considered a more acceptable outcome than failing to prepare for PACF discharge earlier, which could complicate rehabilitation and care coordination, while also increasing potential costs that might have previously been unforeseen for an elective procedure. However, the threshold for the predictive model can be changed depending on the needs of the healthcare team and the desired predictive model characteristics.

Many associations found within this study have been reported in prior literature. The relationship between demographic variables, including increasing age and female gender, with poor discharge outcomes have been previously documented in ASD populations [22]. A higher number of fused interspaces (≥ 8) and regions covering both thoracic and lumbar portions of the spine correspond to increased surgical invasiveness and longer hospital LOS, adverse complications, and poor discharge [23, 24]. However, the negative odds ratio found with revision surgery was unexpected given that revision surgeries are typically associated with greater procedural complications despite similar baseline comorbidities to non-revision patients [25]. One potential explanation is that patients who present for revision operations may be more likely to have support at home or understand the recovery process and needs required following spine surgery given their prior experience. Finally, no studies have previously determined the relationship between institution type (academic or non-academic) and risk of PACF discharge within ASD populations. The finding that academic centers increase likelihood of PACF discharge may be attributable to enhanced access to rehabilitation services and more extensive administrative capacity in supporting transitions.

### Implications

One objective in our use of predictive modeling was to create a parsimonious clinical tool for patient risk assessment. Univariate and multivariate analyses identified several factors as significant predictors of discharge. However, consideration of every single factor and its corresponding odds ratio is often not feasible in the setting of rapid decision-making. Utilization of the LASSO technique for determining variables with the highest importance followed by stepwise logistic regression to assess model accuracy with each successive variable was therefore highly beneficial. Although the fully saturated logistic model using all significant predictors had an accuracy of 0.75, the final simplified logistic model had a similar accuracy of 0.74 and only used seven of the predictors. Therefore, the goal was met in creating a model with both simplicity and retention of accuracy.

Existing predictive analytics in spine surgery have shown substantial benefit. The ACS NSQIP risk calculator and the Risk Assessment Tool (RAT) utilize CPT codes, demographics, and comorbidities to predict hospital LOS, discharge, and medical complications following surgery [26, 27]. While validation studies have evaluated such models as having AUCs between 0.61 and 0.70 [28], none are specific to ASD patients. Moreover, the algorithms were presented with limited transparency on how predictions were generated, which limits their implementation in clinical practice. The benefit of the logistic model presented in this study is that the beta coefficients and recommended cut-off can be readily applied to a calculator and changed as additional data become available with future validation. Furthermore, this study’s usage of solely adult patients who underwent multi-level lumbar/thoracolumbar instrumented fusions is critical for development of a tool to assess risk within the ASD population. Adults with spinal deformity consist of a unique profile of risk factors comorbidities, and clinical presentations compared those with chronic diseases [29]. As such, a predictive model targeted toward this population holds greater validity than existing calculators that are generalized for spine surgery.

Compared to other predictive models that predict discharge outcome in the ASD population, the one presented in this study is one of the first to utilize a large number of patients from multiple healthcare centers (*N* = 8866) to inform development. While other similar models have been previously reported for predicting discharge outcome in ASD patients, few have utilized total populations greater than *N* = 300 [30, 31]. Robust machine learning development requires that samples used to train and validate the model have similar overall characteristics, a goal difficult to achieve with limited sample sizes [32]. Moreover, data derived from a single healthcare institution can result in models that are overly fitted and poorly generalizable to broader healthcare settings. Hence, a key strength of this study is the utilization of a national inpatient database through many institutions.

### Limitations

The results of this study should be considered in the context of its limitations. The first is that the data used were not recently acquired, and hence, predictive models may require future adjustment to reflect current trends. However, the rate of discharge in our study is similar to that of more recent studies following adult spinal deformity surgery, which may suggest that recent changes to pre-operative optimization strategies, intra-operative surgical techniques, peri-operative complication profiles, and post-operative care pathways may not have considerably moved the needle on discharge disposition following deformity operations in adults in the last 10–15 years [8]. Further work is needed to determine if this is truly the case.

As an administrative dataset that relies on ICD codes, our results are reliant on the accuracy of the ICD codes queried, which are commonly not audited. In addition to us not being able to verify the accuracy of the ICD codes, the lack of granularity of the data is a key limitation. For example, while we excluded diagnoses other than spinal deformity, it is possible that patients with purely degenerative pathology were treated in this cohort. As the database only provides umbrella terms for spinal deformities, we are unable to comment on the prevalence of individual diagnoses, severity of deformities (given no radiographic data), and/or granular information on specifics of etiologies of included deformities that compromised our cohort. Furthermore, as the database only groups levels into 3 categories (< 3 levels, 3–7 levels, 8+ levels) and by surgical region, we are not able to comment specifically on how many levels and which levels were treated as well as whether patients had osteotomies (and how many) performed. We are also unable to comment upon what type of surgeries were performed previously for patients who were classified as having undergone a revision operation. Absent from the dataset are information on the condition of the patient before surgery [i.e., ambulatory status, neurological function, and pre-operative living situation (home vs care facility)], which we acknowledge are important contributors to risk of needing post-acute care discharge. Another likely influencer of discharge disposition is peri-operative complications. Although information on some peri-operative complications was available in the dataset, we chose to only evaluate pre-operative factors, as our goal was to build a calculator that could be used pre-operatively to inform discharge location. Another important limitation is the inability to query the dataset for variables that inform patient frailty, distance from treating facility, social support, and socioeconomic status, the latter reportedly being a significant contributor to discharge location. Future studies utilizing metrics to quantify frailty (i.e., sit to stand, 3 min walk), social determinants of health, and social support, including the Risk Assessment and Prediction Tool (RAPT), hold promise in increasing models’ predictive capabilities.

While we acknowledge that the lack of granularity challenges the utility of the results, that our predictive model included a high patient volume from a variety of states was felt to be advantageous for robust machine learning. While granular data can possibly be attained from a single institution, building a predictive model from one institution with a more limited patient cohort size could be overly specific to that singular location and lack broader generalizability. As such, we believe that the predictive model we have identified holds promise for informing and helping patients and families as well as clinicians treating adults with spinal deformity by providing a simple risk assessment tool for discharge disposition. We do also wish for our model to be considered foundational for more granular models that are derived from data sources in the future.

## Conclusion

In this analysis of 8866 adults who underwent multi-level lumbar/thoracolumbar operations for lumbar pathology and spinal deformity, significant variables associated with PACF discharge were age ≥ 60 years, male gender, CCI, COPD, hypertension, hemiplegia/paraplegia, renal disease, drug abuse, osteoporosis, depression, controlled diabetes mellitus, academic institution**,** longer fusions (≥ 8 levels), and private insurance. A simplified predictive model was built using seven selected predictors (insurance, number of interspaces fused/instrumented, gender, age, surgical region, CCI, and revision surgery). With an AUC of 0.74, this model may facilitate identification of adults undergoing elective multi-level lumbar/thoracolumbar spinal instrumented fusions for degenerative pathology and spinal deformity at risk for discharge to a PACF, which may guide early discharge planning and facilitate management of patient and family expectations.

## Supplementary Information

Below is the link to the electronic supplementary material.

Supplementary file1 (DOCX 21 KB)
