# Supplementary material for: Development and internal validation of predictive models to assess risk of post-acute care facility discharge in adults undergoing multi-level instrumented fusions for lumbar degenerative pathology and spinal deformity
Source: Spine Deform. 2022 Sep 20;11(1):163–73. doi: 10.1007/s43390-022-00582-w (PMC9768002; doi:10.1007/s43390-022-00582-w)
Supplement: Supplementary file 1 — Supplementary file1 (DOCX 21 KB) [file 43390_2022_582_MOESM1_ESM.docx]

**Supplementary Table 1.** Beta Coefficients and Equation for Simplified Logistic Predictive Model

| **Logistic Model Component** | **Beta Coefficient** |
| --- | --- |
| *Intercept* | -1.019 |
| Insurance (Private) | -0.549 |
| # Interspaces (8+) | 0.474 |
| Gender (Male) | -0.481 |
| Age* | 0.319 |
| Surgical Region (Lumbar + Thoracic) | 0.496 |
| CCI** | 0.309 |
| Revision Surgery | -0.348 |

* Per decade increase

** Per one point increase

Model Equation:


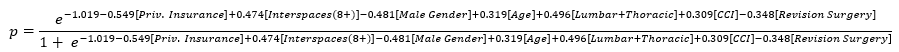


Note for age variable in model equation: Age ranges 50-59=0, 60-69=1, 70-79=2,$\geq$ 80=3.

**Supplementary Table 2.** Diagnosis Codes used for Inclusion Criteria

| **ICD9 Code** | **Description** |
| --- | --- |
| 737.1 | Kyphosis (acquired) |
| 737.2 | Lordosis (acquired) |
| 737.29 | Other Lordosis (acquired) |
| 737.3 | Other idiopathic scoliosis |
| 737.4 | Curvature of spine associated with other conditions |
| 737.8 | Other curvatures of spine |
| 737.9 | Unspecified curvature of spine |
| 738.5 | Other acquired deformity of back or spine |
| 738.9 | Acquired deformity of unspecified site |

**Supplementary Table 3.** Codes used for Patient Exclusion Criteria

| **Description** | **ICD9 Codes or Source of Exclusion Criteria** |
| --- | --- |
| Spine Infection | 324.1, 730.08, 730.18, 730.28, 730.98, 998.59, 998.51, 998.5, 998.13, 998.32, 998.83, 996.63, 996.66, 996.67, 730.00, 730.09, 730.10, 730.19, 730.20, 730.29 |
| Sepsis | 995.91, 038, 038.0, 038.1, 038.10, 038.11, 038.12, 038.19, 038.2, 038.3, 038.4, 038.40, 038.41, 038.42, 038.43, 038.44, 038.49, 038.8, 038.9 |
| Trauma | 805, 806 |
| Metastatic Solid Tumor | 196.0, 196.1, 196.2, 196.3, 196.5, 196.6, 196.8, 196.9, 197.0, 197.1, 197.2, 197.3, 197.4, 197.5, 197.6, 197.7, 197.8, 198.0, 198.1, 198.2, 198.3, 198.4, 198.5, 198.6, 198.7, 198.81, 198.82, 198.89, 199.0, 199.1, 199.2 |
| Any Malignancy Excluding Skin | 140.0,140.1, 140.3, 140.4-140.6, 140.8-141.6, 141.8-142.2, 142.8-143.1, 143.8-144.1, 144.8-145.6, 145.8-147.3, 147.8-148.3, 148.8-149.1, 149.8-150.5, 150.8-151.6, 151.8- 152.3, 152.8-154.3, 154.8, 155.0-155.2, 156.0-156.2, 156.8-157.4, 157.8-158.0, 158.8-159.1, 159.8-160.5, 160.8-161.3, 161.8-162.0, 162.2-162.5, 162.8-163.1, 163.8-164.3, 164.8-165.0, 165.8, 165.9, 170.0-171.0, 171.2-172.9, 174.0-175.0, 175.9-176.5, 176.8, 176.9, 180.0, 180.1, 180.8, 180.9, 18.1, 182.0, 182.1, 182.8, 183.0, 183.2, 183.3, 183.4, 183.5, 183.8, 183.9-184.4, 184.8, 184.9, 185, 186.0, 186.9, 187.1-189.4, 189.8-192.3, 192.8, 192.9, 193, 194.0, 194.1, 194.3-194.6, 194.8-195.5, 195.8, 200.00-200.08, 200.10-200.18, 200.20-200.28, 200.30-200.38, 200.40-200.48, 200.50-200.58, 200.60-200.68, 200.70-200.78, 200.80-200.88, 201.00-201.08, 201.10-201.18, 201.20-201.28, 201.40-201.48, 201.50-201.58, 201.60-201.68, 201.70-201.78, 201.90-201.98, 202.00-202.08, 202.10-202.18, 202.20-202.28, 202.30-202.38, 202.40- 202.48, 202.50-202.58, 202.60- 202.68, 202.70-202.78, 202.80-202.88, 202.90-202.98, 203.00-203.02, 203.10-203.12, 203.80-203.82, 204.00-204.02, 204.10-204.12, 204.20-204.22, 204.80-204.82, 204.90-204.92, 205.00-205.02, 205.10-205.12, 205.20-205.22, 205.30-205.32, 205.80-205.82, 205.90-205.92, 206.00-206.02, 206.10-206.12, 206.20-206.22, 206.80-206.82, 206.90-206.92, 207.00-207.02, 207.10-207.12, 207.20-207.22, 207.80-207.82, 208.00-208.02, 208.10-208.12, 208.20-208.22, 208.80-208.82, 208.90-208.92, 2386 |
| Surgery to Cervical Spine | 81.01, 81.31, 81.02, 81.32, 81.03, 81.33, 84.61, 84.62, 84.66 |
| Non-Elective Procedure | Exclusion of Admission types including: "Trauma", "Urgent", or "Other" |
| Age<50 | Age from Patient Chart |
